# Association of a Single-Nucleotide Variant rs11100494 of the *NPY5R* Gene with Antipsychotic-Induced Metabolic Disorders

**DOI:** 10.3390/pharmaceutics14020222

**Published:** 2022-01-18

**Authors:** Vera S. Dobrodeeva, Natalia A. Shnayder, Maxim A. Novitsky, Azat R. Asadullin, Elena E. Vaiman, Marina M. Petrova, Oleg V. Limankin, Nikolay G. Neznanov, Natalia P. Garganeeva, Regina F. Nasyrova

**Affiliations:** 1 Institute of Personalized Psychiatry and Neurology, Shared Core Facilities, V.M. Bekhterev National Medical Research Centre for Psychiatry and Neurology, 192019 Saint Petersburg, Russia; maximnovitsky93@gmail.com (M.A.N.); droar@yandex.ru (A.R.A.); vaimanelenadoc@gmail.com (E.E.V.); nezn@bekhterev.ru (N.G.N.); 2 Shared Core Facilities “Molecular and Cell Technologies”, V.F. Voino-Yasenetsky Krasnoyarsk State Medical University, 660022 Krasnoyarsk, Russia; stk99@yandex.ru; 3 Department of Psychiatry and Addiction, Bashkir State Medical University, 450008 Ufa, Russia; 4 P.P. Kashchenko Saint Petersburg Psychiatric Hospital No. 1, Gatchinsky District, 190005 Leningrad, Russia; limankin@mail.ru; 5 Department of Psychotherapy and Sexology, I.I. Mechnikov North-Western State University, 191015 Saint Petersburg, Russia; 6 Saint Petersburg Postgraduate Institute of Medical Experts, 194044 Saint Petersburg, Russia; 7 Department of Psychiatry and Addiction, I.M. Pavlov First Saint Petersburg State Medical University, 197022 Saint Petersburg, Russia; 8 Department of General Medical Practice and Polyclinic Therapy, Siberian State Medical University, 634050 Tomsk, Russia; garganeeva@gmail.com; 9 International Centre for Education and Research in Neuropsychiatry, Samara State Medical University, 443099 Samara, Russia

**Keywords:** pharmacogenetics, schizophrenia, NPYR, neuropeptide Y receptor, antipsychotic-induced weight gain, adverse drug reactions

## Abstract

Background: The usage of antipsychotics (APs) is the most robust and scientifically based approach in the treatment of schizophrenia spectrum disorders (SSDs). The efficiency of APs is based on a range of target receptors of the central nervous system (CNS): serotoninergic, dopaminergic, adrenergic, histaminergic and cholinergic. Metabolic disorders are the most severe adverse drug reactions (ADRs) and lead to cardiovascular diseases with a high rate of mortality in patients with SSDs. Neuropeptide Y receptor Y5 (NPY5R) is known in the chain of interaction to target receptors for APs, agouti-related peptide receptors and proopiomelanocortin receptors. We studied the association of the single-nucleotide variants (SNVs) rs11100494 and rs6837793 of the *NPY5R* gene, and rs16147, rs5573, rs5574 of the *NPY* gene, with metabolic disorders in Russian patients with SSDs. Methods: We examined 99 patients with SSDs (mean age—24.56 years old). The mean duration of APs monotherapy was 8 weeks. The biochemical blood test included levels of glucose, cholesterol, lipoproteins, alanine aminotransferase (ALT), aspartate aminotransferase (AST), total protein and albumin. Anthropometry included weight, height, waist circumference and hip circumference. We used real-time PCR to study the carriage of major and minor alleles of the SNV rs11100494 (1164C>A) of the *NPY5R* gene (chromosome localization—4q32.2). Group 1 comprised 25 patients with SSDs taking APs with a change in body weight of more than 6% since the start of APs therapy. Group 2 comprised 74 patients with SSDs taking APs with a change in body weight of less than 6% since the start of APs therapy. Results: We show the significance of genetic risk factors (carriage of major allele C of SNV rs11100494 of the *NPY5R* gene) for the development of AP-induced weight gain in Russian patients with SSDs. The allele C predisposes to AP-induced weight gain (OR = 33.48 [95% CI: 12.62; 88.82], *p*-value < 0.001). Additionally, the results of our study demonstrate that first-generation APs (FGAs) are more likely to cause an increase in serum transaminase levels but are less likely to increase body weight. Second-generation APs (SGAs) are more likely to cause weight gain and changes in serum glucose levels. Conclusion: Our study shows the predictive role of the allele C of SNV rs11100494 of the *NPY5R* gene in the development of AP-induced weight gain. However, we did not find a significant association between biochemical markers and this SNV in Russian patients with SSDs.

## 1. Introduction

Antipsychotics (APs) are the drugs of choice for the treatment of schizophrenia spectrum disorders (SSDs). First-generation APs (FGAs) are high-affinity dopamine D2-receptor antagonists. Second-generation APs (SGAs) have a strong affinity for the serotonin 5-HT_2_ receptor, with concomitant affinities for dopaminergic, muscarinic, histaminergic (H1) and adrenergic (α1, α2) receptors. Therapy for SSDs requires the long-term use of APs, which necessitates a personalized risk assessment for adverse drug reactions (ADRs) [1].

**Figure 1:**
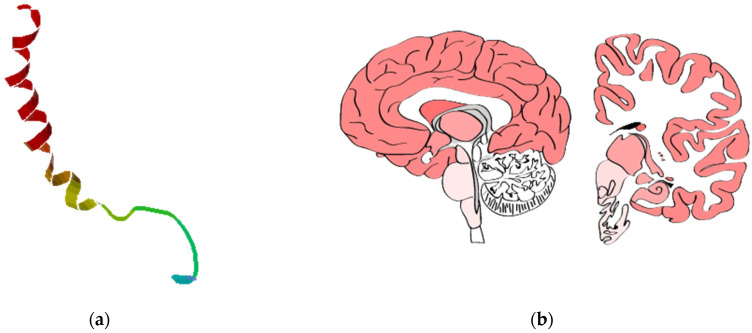

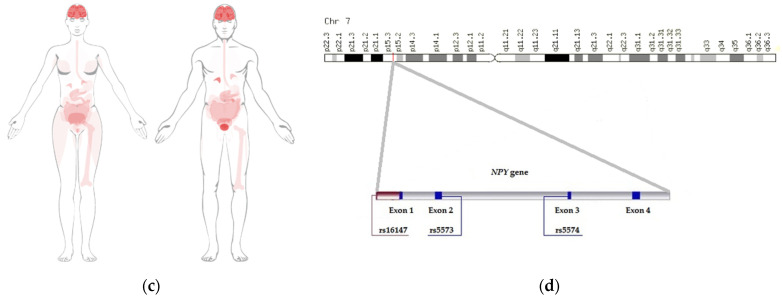
Structure (**a**) and tissue expression (**b**,**c**) of a neuropeptide Y [6]; chromosome location of the *NPY* gene (**d**) [9].

Due to the different mechanisms of action of the FGAs and SGAs, their ADRs are significantly different. FGAs predispose to the development of AP-induced extrapyramidal symptoms, such as tardive dyskinesia and parkinsonism. SGAs have a lower risk of AP-induced extrapyramidal ADRs, but an increased risk of AP-induced weight gain (AIWG) [2]. This substantial AIWG is a leading factor in patient non-compliance and poses a significant risk of diabetes mellitus, lipid abnormalities and cardiovascular disorder, including sudden death syndrome [3]. Patients with SSDs have a 20% shorter lifespan compared with the general population and cardiovascular diseases are the leading cause of death [4]. In addition, AIWG and obesity are major factors contributing to patient non-compliance [5]. The way AIWG develops is yet to be comprehended. The most abundant peptide found in the mammalian brain, neuropeptide Y (NPY), is considered to be one of the most potent orexigenic peptides. NPY is a member of the pancreatic polypeptide (PP-fold) family, which consists of peptide YY (PYY), pancreatic polypeptide (PP) and peptide Y (PY). NPY consists of 36 amino acids, first isolated from the porcine brain in 1982 (Figure 1a) [6]. To gain certain functions, NPY is required to bind to the NPY receptor (NPYR) to activate specific signaling pathways [7]. 

Leptin binding to its hypothalamic receptor regulates the synthesis and release of NPY. NPY links afferents reflecting the nutritional status of the organism from endocrine, gastrointestinal, central (CNS) and peripheral nervous systems to effectors of energy intake and expenditure [8]. Hyperphagia, increases in fat depots, decreases in thermogenesis, and the suppression of sympathetic activity is stimulated by NPY [9].

NPY is encoded by the *NPY* gene. This gene is localized on chromosome 7p15.3 and is approximately 8 kilobases in length, with four exons and three introns (Figure 1d) [10]. The highest expression of NPY is observed in the brain, endocrine tissues, prostate, adipose and soft tissue (Figure 1b,c). The single-nucleotide variants (SNVs) rs16147 (4604T>C) in the promoter region, rs5573 (6203G>A) in exon 2 and rs5574 (10327C>A) in exon 3 of the *NPY* gene are of the most interest for AIWG prediction [11]. 

NPY binds to the G protein Y-coupled Y receptor family, with the highest affinity for the NPY2-R receptor, followed by NPY1-R, NPY5-R, and NPY4-R receptors. Food deprivation increases NPY expression, and this expression has been revealed to be increased in several animal models of obesity, while it is lowered by the action of leptin and insulin on the neurons. Besides the stimulation of food intake, NPY decreases energy expenditure and also induces lipogenesis [12]. The NPY1-R, NPY2-R, NPY4-R and NPY5-R, cloned in the hypothalamus, have all been postulated to mediate the orexigenic effects of NPY [13]. NPY and receptors NPY1-R, NPY2-R, and NPY5-R appear to be involved in the pathophysiology of several diseases including diabetes mellitus, heart failure, arterial hypertension and peripheral arterial disease [14].

NPY5-R is thought to be the main receptor involved in NPY-induced food intake since a reduction in food intake after an injection of antisense oligonucleotides directed against NPY5-R was demonstrated in rats. These results suggest that NPY5-R may be involved in energy balance and is, therefore, a susceptibility candidate gene (the *NPYR* gene) for obesity and related disorders, such as insulin resistance (metabolic) syndrome (IRS) and type 2 diabetes mellitus [15].

**Figure 2:**
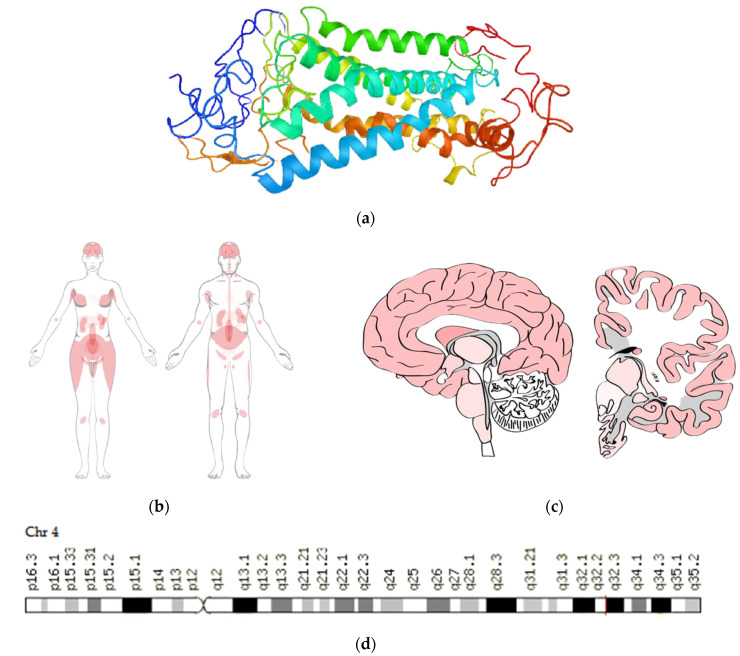
Structure (**a**) and tissue expression (**b**,**c**) of a neuropeptide Y receptor Y5 [6]; chromosome location of the *NPY5R* gene (**d**) [9].

NPY5-R consists of 445 amino acids (Figure 2a) [6] and is encoded by the *NPY5R* gene. *NPY5R* is localized on chromosome 4q32.2 and is approximately 15 kilobases in length, with seven exons and three introns (Figure 2d) [10]. The highest expression of NPY5-R is observed in the brain, bone marrow and lymphoid tissues, adipose and soft tissue (Figure 2b,c). The SNVs rs11100494 (164270253C>A) and rs6837793 (163335583A>G) in the promoter region of the *NPY5R* gene are of the most interest for AIWG prediction [16,17].

## 2. Materials and Methods

This multicenter, observational, open, prospective, randomized pharmacogenetic study was carried out in accordance with the requirements of the Declaration of Helsinki World Medical Association on the ethical principles of conducting medical research involving human subjects (2000) and was approved by the Ethical Committee of V.M. Bekhterev National Medical Research Center for Psychiatry and Neurology (Saint Petersburg, protocol No. 15, dated 18 December 2014). All clinical trials were conducted after the patients signed informed consent and were recorded in accordance with the Ethical Committee of the Russian Federation. Patients gave written informed consent to participate in the clinical and laboratory research. The patients were hospitalized in the Department of First Psychotic Episode in P.P Kashchenko Saint Petersburg Psychiatric Hospital No. 1. and the Republican Clinical Psychiatric Hospital, named after academician V.M. Bekhterev of the Ministry of Healthcare of the Republic of Tatarstan. The inclusion criteria were: Signed informed consent; the age of the patient being 18 to 55 years old; established diagnosis of category F20 according to the International Classification of Diseases 10th revision (ICD-10, 1995); Russian patients of European descent. The exclusion criteria were: Refusal to participate in the study; presence of pronounced somatic pathologies; pregnancy; availability of prior research on APs therapy; non-compliance with AP monotherapy regimen.

The study included 117 patients (95 male and 22 female) permanently residing in the Northwest and Volga Federal Districts of the Russian Federation.

All patients had F20 category mental disorders according to ICD-10. The observation period for our patients averaged 8.36 ± 1.13 weeks. All patients received the same diet: three meals per day, 2200–2400 kcal/day. Patients did not receive additional food sources.

The randomization was carried out by the inclusion and exclusion methods and the random numbers generation method. At the stage of randomization and visits, some patients dropped out of this study due to a change in the treatment regimen (transfer from monotherapy to polytherapy of Aps) or refusal to follow the protocol of this study.

During the study, two visits were conducted: The 1st visit, at the moment of the randomization and inclusion of the patients in the study; the 2nd visit, at the end of the observation period. Anthropometric characteristics (weight, height, waist circumference, thigh circumference) were measured, and clinical and biochemical blood tests were conducted at each visit. Blood samples for genetic testing were drawn during the 1st visit (Table 1).

During the study, 40 patients took FGAs and 76 patients took SGAs. Based on the obtained therapy, two groups were allocated: group 1—patients taking FGAs; group 2—patients taking SGAs. In the observation groups, two subgroups were allocated according to the criterion of change in body weight while taking APs: subgroup 1—change in body weight of more than 6% of the original; subgroup 2—change in body weight of less than 6% from the start of APs therapy.

Biological material was taken in the morning, on an empty stomach.

Blood biochemical parameters included the following markers: glucose; cholesterol; triglycerides; low-density lipoproteins (LDL); high-density lipoproteins (HDL); alanine aminotransferase (ALT); aspartate aminotransferase (AST); total protein; and albumin. Biochemical markers were detected using reagent kits «Glyukoza-UF-Novo», «Holesterin-Novo», «LVP-holesterin-Novo-A» («Vektor-BEST», Russia).

During the 2nd visit, we performed genetic testing. Samples for genetic analysis were stored at a temperature of −78 degrees Celsius. DNA extraction kit was «RIBO-prep» («AmpliTest», Russia). The SNVs in the *NPY* and *NPY5R* genes were selected from the literature and from public databases, such as NCBI (http://www.ncbi.nlm.nih.gov/SNP, 14 August 2015). Genotyping was performed by real-time PCR (PCR RT). All primers were developed by “The Central Research Institute for Epidemiology” of the Federal Service for Surveillance on Consumer Rights Protection and Human Wellbeing (Moscow, Russia).

Statistical analysis was carried out using the programming language R and its library packages (dplyr, ggplot2, psych, ROCR), and the programs for statistical analysis LePac, Arlequin 3.5, and Genepop. The Shapiro–Wilk test was used to test the normality of the distribution of the quantitative variables. Quantitative traits between two groups were compared using a paired T-test subject to the normal distribution of the trait in the groups; in violation of the normality condition, the paired Wilcoxon test was used.

Differences in quality characteristics between the groups were established using the Chi-square test and Fisher’s exact test. The correlation coefficient was established according to Spearman’s criterion. Differences between four or more subgroups were established by analysis of variance, corresponding with the studied sign of the distribution normality and dispersion homogeneity; in case of the violation of compliance, the Kruskal criterion and post hoc Dunne test with Bonferroni’s amendment were used. For the magnitude of the effect of carriage of a particular allele on body weight change, odds ratio (OR) was calculated, and the risk value was measured using a risk ratio (RR). For the definition of the confidence interval (CI), the classical approximation approach and the base approach were used. The level of significance in all tests was taken at 0.05.

## 3. Results

The study included 117 Russian patients of European descent—95 male and 22 female—permanently residing in the Northwest and Volga Federal Districts of the Russian Federation. All patients had F2 category mental disorders according to ICD-10: F20—93; F20.2—1; F20.6—3; F20 + F10.2—2; F21.8—2; F22.8—3; F23—4; F23.1—4; F25.1—5. The mean age of mental disorder was 24.56 ± 1.95 years; the mean age of circulation for medical care was 26.5 ± 1.65 years; the mean age of starting APs therapy was 25.7 ± 1.7 years. The observation period for our patients averaged 8.36 ± 1.13 weeks.

A total of 41 patients took FGAs: 28—haloperidol, six—zuclopenthixol, six—trifluoperazine, one—aminazin; 76 patients took SGAs: 16—risperidone, 15—olanzapine, 12—quetiapine, 11—clozapine, five—asenapine, five—sertindole, four—paliperidone, three—aripiprazole, two—xeplion, one—sulpiride, one—amisulpiride, and one—resperidone (Table 2). Haloperidol (68.3%) was the most prescribed FGAs; risperidone (21.1%) and olanzapine (19.7%) were the most prescribed SGAs.

During the study, 18 patients changed therapy or dropped out of the study for other reasons. Based on the obtained therapy, two groups were allocated out of 99 patients: group 1—patients taking FGAs; group 2—patients taking SGAs. In the observation groups, two subgroups were allocated according to the criterion of change in body weight while taking APs: subgroup 1—change in body weight of more than 6% from the original; subgroup 2—change in body weight of less than 6% from the start of APs therapy (Table 3).

The distribution of the genotypes of SNVs rs11100494 and rs6837793 of the *NPY5R* gene, and rs16147, rs5573 and rs5574 of the *NPY* gene in the first and second observation groups did not have statistically significant differences (*p* > 0.05) (Table 4).

The analysis of the effect of the carriage of certain alleles on changes in body weight was carried out by calculating OR. We used allelic analysis and recessive comparison model. Significant results were revealed for the SNV rs11100494 in the *NPY5R* gene. The analysis was carried out in two versions: against recessive homozygotes and allelic. The C allele of rs11100494 predisposes patients to AIWG over 6%: OR = 33.48, CI = [12.62; 88.82], *p*-value < 0.001. The A allele rs11100494 has a protective effect on this change: OR = 0.03, CI = [0.01; 0.08], *p*-value < 0.05.

## 4. Discussion

The results obtained allow us to conclude that the groups of patients formed by the type of APs did not differ statistically significantly in terms of age and sex characteristics. At the time of study entry, participants taking SGAs were not statistically significantly different from participants taking FGAs in terms of the markers of metabolic syndrome—waist circumference and fasting glucose concentration—measured. Additionally, no differences were found in the distribution of SNV genotypes between these two groups, allowing us to conclude that these two groups are comparable.

Compliance with the Hardy–Weinberg distribution law allows us to draw a conclusion about the genetic representativeness of the sample, which allows us to extend the results obtained to the general population.

During the entire observation period, the patients were under inpatient observation, thereby minimizing differences in nutrition and lifestyle. It can be assumed that any further differences in patients were caused using APs and can be explained by them, as well as by the genetic characteristics of the patients. APs do not have a direct effect on the NPY and NPY5R, but they introduce an imbalance in the central and peripheral mechanisms of regulation of the patient’s metabolism and eating behavior.

The severity of these disorders largely depends on the genetic profile of the patients, which currently cannot be corrected, unlike other factors. Knowledge of the mechanisms at all levels (pharmacokinetics and pharmacodynamics of APs, genetic factors of major metabolic disorders, intracellular and extracellular biochemical markers, food preferences and patient lifestyles) is essential for the selection of safe and effective therapeutic practice in the treatment of mental disorders [18].

The obtained result demonstrates the need for further study of this polymorphism in the context of AP-induced metabolic disorders. A 2007 study at the Institute of Life (Hart-ford, CT, USA) and three hospitals in Kentucky (Lexington, KY, USA) [13] did not show an association between SNV rs11100494 in the *NPY5R* gene and weight gain with olanzapine and risperidone. However, it was reported that SNV rs6837793 in the *NPY5R* gene was associated with weight gain in risperidone-treated (*p* = 0.0024) but not in olanzapine-treated patients. The discrepancy in results may be due to ethnic differences in the sample or different study designs.

An increase in the sample size and the study of the prevalence of this polymorphism in different regions of the Russian Federation and in different populations will provide a more robust result. However, the accumulated data allow us to consider this polymorphism as a candidate for a pharmacogenetic panel. The development of this diagnostic panel is an important step towards the transition to a personalized approach to APs therapy, and therefore, will lead to an increase in the effectiveness and safety of therapy, allowing a reduction in the length of the patient’s stay in the hospital, prescription of the optimal dose of the APs, improvement in survival prognosis, and compliance with the APs used [19].

## 5. Limitations

The limitation of this study is the small sample size and short follow-up period (8 weeks). It is possible that an increase in the follow-up period (>8 weeks) may lead to an increase in the number of patients with AIWG. In addition, Russia is a multinational country with ethnic and racial diversity, which makes it impossible to extrapolate the results to the entire population of Russia.

The expression levels of NPY and NPY5R were not measured in this study, since they were not part of the goal of this stage of research. We hypothesize that the SNV rs11100494 of the *NPY5R* gene is associated with AP-induced weight gain based on the data obtained from statistical analysis. We believe that SNV rs11100494 may be promising for further study in relation to AP-induced metabolic disorders.

However, conducting research in the future and planning multicenter studies in Russia can answer the question: do ethnicity and race influence the association of the C allele of SNV rs11100494 of the *NPY5R* gene with the development of AIWG?

## 6. Conclusions

The results of our study demonstrate the statistical significance of the association of the major C allele of SNV rs11100494 of the *NPY5R* gene with a risk of >6% weight gain in patients with SSDs, regardless of the type of APs taken.

The data obtained indicate the possible isolation of patients with AP-induced metabolic disorders (weight gain) at genetic risk, and the modification of the plan and the duration of laboratory screening of these ADRs, depending on the personal genetic profile.

## Figures and Tables

**Table 1:** Study design.

Parameters	Randomized (2 Weeks) 577 Patients	1st Visit 117 Patients	2nd Visit (After 8 Weeks) 99 Patients
Consultation with a psychiatrist			
Assessment of positive and negative symptoms	+	(-)	(-)
Analysis of ADRs	+	+	+
Analysis vita	+	(-)	(-)
Medical history	+	(-)	(-)
Treatment regimen analysis	+	+	+
Pedigree analysis	+	(-)	(-)
Diet History Questionnaire (DHQ)	+	(-)	(-)
Food Frequency Questionnaire (FFQ)	+	(-)	(-)
Anthropometric data			
Weight, kg	(-)	+	+
Height, cm	(-)	+	+
Waist circumference, cm	(-)	+	+
Thigh circumference, cm	(-)	+	+
Clinical and biochemical blood analysis			
Glucose, mmol/L	(-)	+	+
Cholesterol, mmol/L	(-)	+	+
Triglycerides, mmol/L	(-)	+	+
Low-density lipoproteins (LDL), mmol/L	(-)	+	+
High-density lipoproteins (HDL), mmol/L	(-)	+	+
Alanine aminotransferase (ALT), units per liter	(-)	+	+
Aspartate aminotransferase (AST), units per liter	(-)	+	+
Total protein, g/L	(-)	+	+
Albumin, g/L	(-)	+	+
Pharmacogenetic testing			
Blood sampling	(-)	+	(-)
DNA testing	(-)	+	(-)
Pharmacogenetic analysis	(-)	(-)	+

**Table 2:** Frequency of antipsychotics taken by patients during the study.

FGAs		SGAs	
APs	Number of Patients (%)	APs	Number of Patients (%)
Haloperidol	28 (68.3)	Risperidone	16 (21.1)
Zuclopenthixol	6 (14.6)	Olanzapine	15 (19.7)
Trifluoperazine	6 (14.6)	Quetiapine	12 (15.8)
Aminazine	1 (2.4)	Clozapine	11 (14.5)
		Azenapine	5 (6.6)
		Sertindole	5 (6.6)
		Paliperidone	4 (5.3)
		Aripiprazole	3 (3.9)
		Xeplion	2 (2.6)
		Sulpiride	1 (1.3)
		Amisulpride	1 (1.3)
		Risperidone	1 (1.3)

**Table 3:** Groups of patients with schizophrenia spectrum disorders.

Group, Subgroup	Mean Age (Years Old)	Gender (Male/Female)	Mean Duration of SSDs (Years)
Group 1, subgroup 1 n = 8	33.37 ± 8.48	5/3	5.68 ± 1.57
Group 1, subgroup 2 n = 28	34.14 ± 4.22	20/8	7.28 ± 1.3
Group 2, subgroup 1 n = 17	29.00 ± 4.08	16/1	8.41 ± 2.06
Group 2, subgroup 2 n = 46	35.06 ± 3.02	39/7	9.25 ± 2.4

**Table 4:** Distribution of genotypes for studied SNVs in observation groups of patients with schizophrenia spectrum disorders.

**Group**	**Genotypes SNV (rs11100494) in** **the *NPY5R* Gene, n**			**Allele A, n**	**Allele G, n**
	**AA**	**AG**	**GG**		
Group 1	0	7	34	7	75
Group 2	1	4	66	6	136
F-test, *p* value = 0.09					
Measurement of exact *p* value of conformity to Hardy–Weinberg’s law by Markov chain method					
Parameters	*p*.val	S.E	W&C	R&H	Steps
General compliance	0.08	0.00	0.19	0.19	43,032
Deficiency of heterozygotes	0.09	0.00	0.19	0.19	43,032
Excess heterozygotes	0.99	0.00	0.19	0.19	43,032
**Group**	**Genotypes SNV (rs6837793) in** **the *NPY5R* gene, n**			**Allele A, n**	**Allele G, n**
	**AA**	**AG**	**GG**		
Group 1	0	7	33	7	73
Group 2	0	14	57	14	128
F-test, *p* value = 0.99					
Measurement of exact *p* value of conformity to Hardy–Weinberg’s law by Markov chain method					
Parameters	*p*.val	S.E	W&C	R&H	Steps
General compliance	1.00	0.00	−0.02	−0.02	60,754
Deficiency of heterozygotes	0.74	0.00	−0.02	−0.02	60,754
Excess heterozygotes	0.64	0.00	−0.02	−0.02	60,754
**Group**	**Genotypes SNV (rs16147) in** **the *NPY* gene, n**			**Allele C, n**	**Allele T, n**
	**CC**	**CT**	**TT**		
Group 1	9	21	11	39	42
Group 2	17	27	27	61	81
F-test, *p* value = 0.37					
Measurement of exact *p* value of conformity to Hardy–Weinberg’s law by Markov chain method					
Parameters	*p*.val	S.E	W&C	R&H	Steps
General compliance	0.13	0.00	0.14	0.14	84,983
Deficiency of heterozygotes	0.09	0.00	0.14	0.14	84,983
Excess heterozygotes	0.95	0.00	0.14	0.14	84,983
**Group**	**Genotypes SNV (rs5573) in** **the *NPY* gene, n**			**Allele A, n**	**Allele G, n**
	**AA**	**AG**	**GG**		
Group 1	6	4	31	16	66
Group 2	8	9	53	25	115
F-test, *p* value = 0.84					
Measurement of exact *p* value of conformity to Hardy–Weinberg’s law by Markov chain method					
Parameters	*p*.val	S.E	W&C	R&H	Steps
General compliance	0.00	0.00	0.60	0.61	76,065
Deficiency of heterozygotes	0.00	0.00	0.60	0.61	76,065
Excess heterozygotes	1.00	0.00	0.60	0.61	76,065
**Group**	**Genotypes SNV (rs5574) in** **the *NPY* gene, n**			**Allele C, n**	**Allele T, n**
	**CC**	**CT**	**TT**		
Group 1	14	24	3	52	30
Group 2	29	29	13	87	55
F-test, *p* value = 0.12					
Measurement of exact *p* value of conformity to Hardy–Weinberg’s law by Markov chain method					
Parameters	*p*.val	S.E	W&C	R&H	Steps
General compliance	1.00	0.00	0.01	0.01	84,293
Deficiency of heterozygotes	0.52	0.00	0.01	0.01	84,293
Excess heterozygotes	0.62	0.00	0.01	0.01	84,293
